# Role of Granulocyte-Macrophage Colony-Stimulating Factor Production by T Cells during *Mycobacterium tuberculosis* Infection

**DOI:** 10.1128/mBio.01514-17

**Published:** 2017-10-24

**Authors:** Alissa C. Rothchild, Britni Stowell, Girija Goyal, Cláudio Nunes-Alves, Qianting Yang, Kadamba Papavinasasundaram, Christopher M. Sassetti, Glenn Dranoff, Xinchun Chen, Jinhee Lee, Samuel M. Behar

**Affiliations:** a Program in Immunology, Division of Medical Sciences, Harvard Medical School, Boston, Massachusetts, USA; b Department of Microbiology and Physiological Systems, University of Massachusetts Medical School, Worcester, Massachusetts, USA; c Department of Medical Oncology, Dana-Farber Cancer Institute, and Department of Medicine, Brigham and Women’s Hospital and Harvard Medical School, Boston, Massachusetts, USA; d Guangdong Key Laboratory for Emerging Infectious Diseases, Shenzhen Key Laboratory of Infection and Immunity, Shenzhen Third People’s Hospital, Shenzhen, China; e Department of Pathogen Biology, Shenzhen University School of Medicine, Shenzhen, China; Albert Einstein College of Medicine

**Keywords:** GM-CSF, *Mycobacterium tuberculosis*, T cells, cytokines, lung infection, macrophages

## Abstract

Mice deficient for granulocyte-macrophage colony-stimulating factor (GM-CSF^−/−^) are highly susceptible to infection with *Mycobacterium tuberculosis*, and clinical data have shown that anti-GM-CSF neutralizing antibodies can lead to increased susceptibility to tuberculosis in otherwise healthy people. GM-CSF activates human and murine macrophages to inhibit intracellular *M. tuberculosis* growth. We have previously shown that GM-CSF produced by iNKT cells inhibits growth of *M. tuberculosis*. However, the more general role of T cell-derived GM-CSF during infection has not been defined and how GM-CSF activates macrophages to inhibit bacterial growth is unknown. Here we demonstrate that, in addition to nonconventional T cells, conventional T cells also produce GM-CSF during *M. tuberculosis* infection. Early during infection, nonconventional iNKT cells and γδ T cells are the main source of GM-CSF, a role subsequently assumed by conventional CD4^+^ T cells as the infection progresses. *M. tuberculosis*-specific T cells producing GM-CSF are also detected in the peripheral blood of infected people. Under conditions where nonhematopoietic production of GM-CSF is deficient, T cell production of GM-CSF is protective and required for control of *M. tuberculosis* infection. However, GM-CSF is not required for T cell-mediated protection in settings where GM-CSF is produced by other cell types. Finally, using an *in vitro* macrophage infection model, we demonstrate that GM-CSF inhibition of *M. tuberculosis* growth requires the expression of peroxisome proliferator-activated receptor gamma (PPARγ). Thus, we identified GM-CSF production as a novel T cell effector function. These findings suggest that a strategy augmenting T cell production of GM-CSF could enhance host resistance against *M. tuberculosis*.

## IMPORTANCE

*Mycobacterium tuberculosis* is the bacterium that causes tuberculosis, the leading cause of death by any infection worldwide. T cells are critical components of the immune response to *Mycobacterium tuberculosis*. While gamma interferon (IFN-γ) is a key effector function of T cells during infection, a failed phase IIb clinical trial and other studies have revealed that IFN-γ production alone is not sufficient to control *M. tuberculosis*. In this study, we demonstrate that CD4^+^, CD8^+^, and nonconventional T cells produce GM-CSF during *Mycobacterium tuberculosis* infection in mice and in the peripheral blood of infected humans. Under conditions where other sources of GM-CSF are absent, T cell production of GM-CSF is protective and is required for control of infection. GM-CSF activation of macrophages to limit bacterial growth requires host expression of the transcription factor PPARγ. The identification of GM-CSF production as a T cell effector function may inform future host-directed therapy or vaccine designs.

## INTRODUCTION

*Mycobacterium tuberculosis*, the causative agent of tuberculosis (TB), is a bacterium that latently infects nearly one-third of the world’s population, causing active disease in 10% of individuals (1). Bacillus Calmette-Guérin (BCG), the only approved vaccine available against TB, has variable efficacy in different populations, and it is generally agreed that a better vaccine is needed (2). Thus, it is crucial to identify protective immune mechanisms against *M. tuberculosis* that might help in the design of future therapeutics or vaccines.

More than three decades ago, it was demonstrated that CD4^+^ and CD8^+^ T cells were required for an effective immune response to *M. tuberculosis* (3–5). Similarly, gamma interferon (IFN-γ) was identified as a key cytokine that leads to inhibition and killing of *M. tuberculosis* through induction of nitric oxide (6), phagolysosomal fusion (7), autophagy (8), and vitamin D receptor expression (9), although T cell production of IFN-γ has only recently been linked to *M. tuberculosis* control (10). Despite the central role of IFN-γ, there is evidence that too much IFN-γ may be detrimental and that T cells can control *M. tuberculosis* growth *in vivo* independently of IFN-γ (11–13). The role of IFN-γ in people is more difficult to discern. Importantly, a phase IIb clinical trial testing a heterologous prime-boost strategy of BCG followed by a modified vaccinia Ankara virus expressing *M. tuberculosis* antigen 85 (MVA85) increased IFN-γ production by antigen-specific T cells but had no impact on protection from *M. tuberculosis* infection over 2 years (14). Collectively, these data suggest that T cell-mediated mechanisms of protection other than IFN-γ production may mediate protection.

Apart from IFN-γ production, there are several other T cell effector functions that contribute to an antimicrobial response. Mice lacking tumor necrosis factor (TNF) are highly susceptible to *M. tuberculosis* infection (15), and T cell production of TNF has been shown to be critical for protection (16). Interleukin-17 (IL-17) has also been shown to have an important role in controlling *M. tuberculosis* and in effective granuloma formation during the early phase of infection, but too much IL-17 can have detrimental effects by promoting immunopathology (11, 17, 18). In addition, cytolytic activity by CD8^+^ T cells has been shown to contribute to protection in both mice and humans (19–22). Identifying additional effector cytokines produced by T cells during *M. tuberculosis* infection could provide novel immunotherapy targets and new potential correlates of protection for vaccine evaluation.

We previously showed that iNKT cell production of granulocyte-macrophage colony-stimulating factor (GM-CSF) contributes to their ability to restrict bacterial growth *in vitro* and that GM-CSF treatment of macrophages restricts *M. tuberculosis* growth in murine cells (23). GM-CSF treatment of human macrophages inhibits intracellular growth of *M. tuberculosis* and *Mycobacterium avium* (24–26), and GM-CSF^−/−^ mice are highly susceptible to *M. tuberculosis* (27, 28). However, GM-CSF production is not restricted to iNKT cells; it is produced by many different cell types, including leukocytes (29, 30), epithelial cells (31), and fibroblasts (32), and it was originally identified for its role in maturation of both macrophages and granulocytes from bone marrow precursor cells *in vitro* (33). GM-CSF is commonly used to differentiate dendritic cells *in vitro* (34), and it is used clinically to boost myeloid recovery after chemotherapy. Mice lacking GM-CSF develop normally and show no signs of abnormal steady-state hematopoiesis (35, 36). However, these mice do have dramatic defects in lung function, which can be explained by a failure of alveolar macrophages to develop (37, 38). An absence of GM-CSF leads to dysregulation of surfactant recycling in alveolar macrophages, generating a pulmonary inflammatory defect that resembles the human disease pulmonary alveolar proteinosis (PAP). Overexpression of GM-CSF by type II epithelial cells after insertion of a GM-CSF transgene on a surfactant protein C promoter abrogated the development of PAP in GM-CSF^−/−^ mice (39). These and other studies led to the idea that pulmonary epithelial cells are the main producers of GM-CSF in the lung, and GM-CSF is important for normal lung homeostasis. Interestingly, overexpression of GM-CSF in epithelial cells in GM-CSF^−/−^ mice led to only partial rescue of *M. tuberculosis* susceptibility, suggesting that GM-CSF production by other cells may also contribute to protection (27, 28). Additionally, clinical studies have found that the presence of anti-GM-CSF autoantibodies that block GM-CSF function are associated with susceptibility to both cryptococcal meningitis and pulmonary TB in otherwise healthy subjects (40), indicating that GM-CSF may also participate in host defense against infection in people. In contrast, immunotherapy that induces GM-CSF production was recently shown to be effective against both drug-resistant and drug-sensitive *M. tuberculosis* infection in mice (41, 42).

Here, we tested the hypothesis that T cell production of GM-CSF contributes to host resistance to *M. tuberculosis*. We found that numerous T cell subsets, including both conventional and unconventional T cells, produce GM-CSF after infection. T cell production of GM-CSF was not required for the T cell ability to transfer protection to susceptible hosts when other cell types already produced GM-CSF. However, in the absence of other sources of GM-CSF, GM-CSF production by hematopoietic cells and specifically T cells can contribute to host resistance. GM-CSF is produced by human T cells and can have additive protective effects with IFN-γ toward *M. tuberculosis*-infected macrophages. While GM-CSF induces numerous changes to mature macrophages, we discovered that GM-CSF control of *M. tuberculosis* growth requires peroxisome proliferator-activated receptor gamma (PPARγ) expression. PPARγ is a nuclear receptor that controls cellular lipid and glucose metabolism, and regulation of PPARγ by GM-CSF has been shown to be essential for alveolar macrophage development and surfactant homeostasis in the lung (38, 43). These results show that T cell production of GM-CSF contributes to control of *M. tuberculosis* infection in the absence of other sources of GM-CSF, that multiple T cell subsets make GM-CSF in the lung over the course of infection, and that GM-CSF can act directly on infected macrophages through a pathway requiring PPARγ to limit bacterial growth.

## RESULTS

### GM-CSF production is increased in the lungs during *M. tuberculosis* infection.

**FIG 1:**
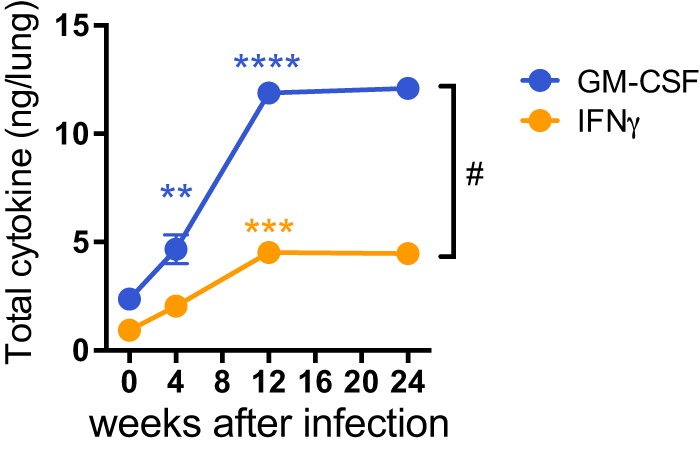
GM-CSF production in the lung increases over the course of *M. tuberculosis* infection. GM-CSF was measured in a Bioplex immunoassay in lung homogenates at certain weeks post-aerosol infection with the Erdman strain in WT C57BL/6J mice. GM-CSF and IFN-γ protein levels were normalized per lung. **, *P* < 0.01; ***, *P* < 0.001; ****, *P* < 0.0001 (compared to previous time point). #, *P* < 0.05 for GM-CSF versus IFN-γ.

To determine the overall production of GM-CSF in the lung during *M. tuberculosis* infection, we measured GM-CSF concentrations in lung homogenates. A small amount of GM-CSF was detected in the lungs of uninfected mice, and the amount increased following *M. tuberculosis* infection (Fig. 1). The kinetics of GM-CSF accumulation in the lungs of *M. tuberculosis*-infected mice roughly coincided with the development of T cell immunity and inflammatory changes in the lung, and it paralleled IFN-γ production, a cytokine predominantly made by T cells (44). Of note, GM-CSF was more abundant than IFN-γ, which is one of the classic markers of T cell immunity to *M. tuberculosis*. The increased levels of GM-CSF persisted in the lung during chronic *M. tuberculosis*, with an increase of 2.0-fold ± 0.3-fold (mean ± standard error of the mean [SEM]) at 4 weeks, 5.0-fold ± 0.2-fold at 12 weeks, and 5.1-fold ± 0.1-fold at 24 weeks above that in lungs of uninfected mice. These data confirmed that GM-CSF production accumulates in the lung over the course of infection (45), and the kinetics suggest that its production increases as an adaptive immune response develops in the lungs.

### GM-CSF production by both radioresistant and radiosensitive cells contributes to host resistance against TB.

**FIG 2:**
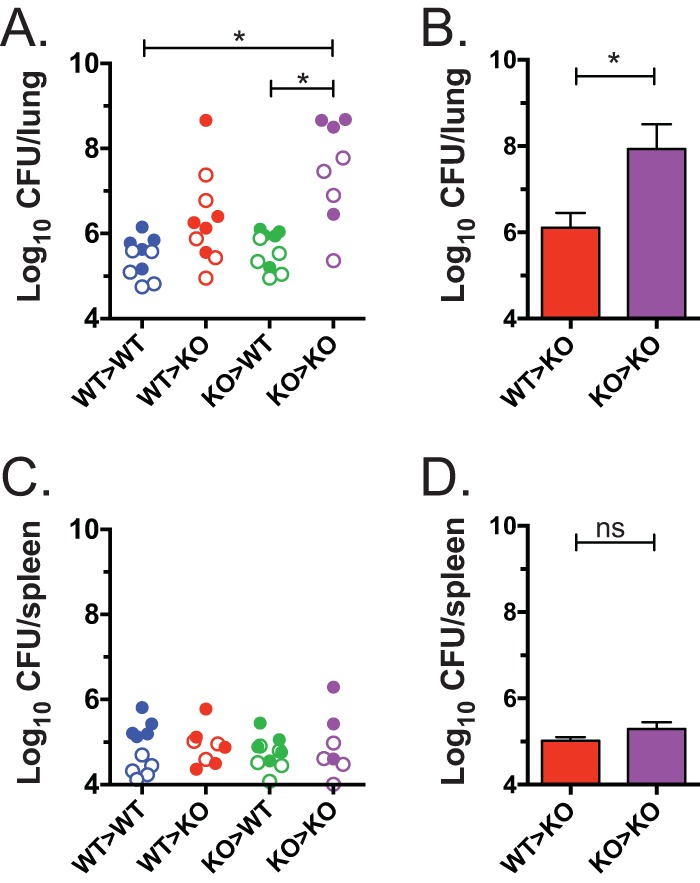
GM-CSF production by either radioresistant or radiosensitive cells promotes mycobacterial control. (A and C) Reciprocal radiation chimeric mice (donor BM → recipient) were allowed to reconstitute for 8 weeks and then challenged with aerosolized *M. tuberculosis*, and lung (A) and spleen (C) CFU were determined after 4 weeks. Each group contained 4 to 5 mice, and data were combined from 2 independent experiments (results of the first experiment are shown as open symbols, and those from the second experiment are shown as closed symbols). A one-way ANOVA was used. *, *P* < 0.05. See Table S1 for statistical analysis of each experiment’s data, analyzed separately and also combined. (B and D) A third experiment was done to compare the how WT → KO and KO → KO BM chimeras controlled *M. tuberculosis* infection in the lungs (B) and spleen (D). The groups contained 7 and 5 mice, respectively. Bars represent means ± SEM. Analysis was performed using an unpaired *t* test. *, *P* < 0.05 (WT versus C57BL/6 mice and KO versus GM-CSF^−/−^ mice).

In previous studies, the susceptibility of GM-CSF^−/−^ mice to *M. tuberculosis* was shown to be partially rescued if GM-CSF was overexpressed under the control of the surfactant protein C promoter, a gene thought to be specific for type II pulmonary epithelial cells (27, 28). Under those conditions, survival was prolonged but not completely rescued. Therefore, we hypothesized that additional cell types could be important and necessary sources of GM-CSF during TB. Specifically, we considered whether GM-CSF production by epithelial cells and other nonhematopoietic cells (generally radioresistant) was sufficient for host defense against *M. tuberculosis* or whether hematopoietic cells (generally radiosensitive) were also required. To test this hypothesis, reciprocal radiation chimeric mice using wild-type (WT) and GM-CSF^−/−^ donor bone marrow (BM) and recipient mice were produced. Transferring GM-CSF^−/−^ BM into GM-CSF^−/−^ recipients (KO → KO) resulted in chimeric mice that were more susceptible than chimeric mice in which WT BM was transferred to WT recipient mice (WT → WT), based on their lung bacterial burden 4 weeks after *M. tuberculosis* challenge (Fig. 2A). Chimeras made by transferring GM-CSF^−/−^ BM into WT recipients (KO → WT) were similarly resistant as WT → WT chimeric mice, indicating that GM-CSF production by radioresistant cells was sufficient to confer host resistance, which is consistent with the established role for GM-CSF production by pulmonary epithelial cells. Chimeric mice generated by injecting WT BM into GM-CSF^−/−^ recipients (WT → KO) trended toward being more resistant than the KO → KO mice in two independent experiments, although this result was not statistically significant. When the experiment was repeated with greater statistical power (*n* = 5 to 7 mice/group), the WT → KO chimeras controlled *M. tuberculosis* lung infection better than KO → KO chimeras (*P* < 0.05) (Fig. 2B). Of note, there were no differences in bacterial control within the spleen between experimental groups (Fig. 2C and D). These results showed that GM-CSF is required for control of *M. tuberculosis* in the lungs but is dispensable for control in the spleen (27). Importantly, these results indicate that GM-CSF produced by hematopoietic cells can contribute to resistance against *M. tuberculosis*.

### Multiple T cell subsets produce GM-CSF during *M. tuberculosis* infection.

**FIG 3:**
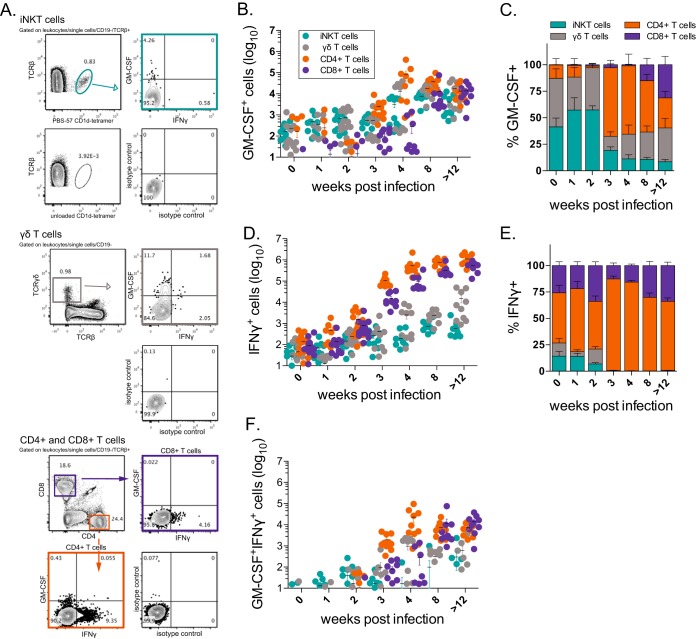
Multiple T cell subsets produce GM-CSF in the lung during *M. tuberculosis* infection. (A) Representative flow cytometry plots after gating of iNKT cells, γδ T cells, and CD4^+^ and CD8^+^ T cells. Controls for CD1d tetramer and GM-CSF and IFN-γ ICS staining are included. (B and C) Absolute cell numbers (B) and the relative frequency of GM-CSF^+^ T cells in C57BL/6J mice at various time points after aerosol infection with *M. tuberculosis* Erdman (C). (D and E) Absolute cell numbers (D) and relative frequencies of IFN-γ^+^ T cells (E). (F) Absolute numbers of GM-CSF^+^ IFN-γ^+^ T cells. Each point represents the result for an individual mouse. Lung cells were cultured in brefeldin A without additional stimulation, and then intracellular cytokine staining was performed. iNKT cells (TCR-β^+^ CD1d tetramer^+^), γδ T cells (CD3^+^ TCR-β^−^ TCR-γδ^+^), CD4^+^ (TCR-β^−^ CD4^+^ CD8^−^), and CD8^+^ (TCR-β^−^ CD4^−^ CD8^+^) were evaluated. Data were compiled from 2 or 3 independent experiments for each time point.

To directly test whether T cells produce GM-CSF during *M. tuberculosis* infection, we performed intracellular cytokine staining (ICS) analysis of lung samples of uninfected or *M. tuberculosis*-infected mice at multiple time points. To determine which cells were actively secreting GM-CSF in the lung, rather than detecting the cells capable of secreting the cytokine, ICS was performed in the presence of IL-2 and brefeldin A without further antigen exposure or other stimulation. Separate staining panels were used to assess the four major T cell subsets, which were gated following doublet and autofluorescence exclusion: iNKT cells (T cell receptor β^+^ [TCR-β^+^] CD1d tetramer^+^), γδ T cells (CD3^+^ TCR-β^−^ TCR-γδ^+^), CD4^+^ T cells (TCR-β^+^ CD4^+^ CD8^−^), and CD8^+^ T cells (TCR-β^+^ CD4^−^ CD8^+^) (Fig. 3A). We found that all 4 subsets produced GM-CSF during infection (Fig. 3B). The relative proportions of each of the T cell subsets producing GM-CSF at each time point were calculated. A small but detectable number of iNKT cells and γδ T cells produced GM-CSF early during infection (0 to 2 weeks) (Fig. 3C). CD4^+^ T cells began to dominate the GM-CSF response between weeks 3 and 4, while CD8^+^ T cells were not a significant source of GM-CSF until week 8 postinfection. IFN-γ production by these subsets was concurrently assessed for comparison (Fig. 3D). In contrast to GM-CSF, CD4^+^ T cells were clearly the dominant IFN-γ producers at most time points (Fig. 3E). Dual GM-CSF/IFN-γ-producing cells were not detected until week 3 and were mostly CD4^+^ and CD8^+^ T cells (Fig. 3F).

These data showed that both innate-like and adaptive T cell subsets produce GM-CSF in the lung in response to *M. tuberculosis* infection. All four T cell subsets displayed statistically significant increases in GM-CSF production during infection compared to baseline (week 0, uninfected) (iNKT cells and γδ T cells, *P* < 0.0001; CD4^+^ T cells, *P*  = 0.0006; CD8^+^ T cells, *P*  = 0.0001; one-way analysis of variance [ANOVA] with Dunnett’s posttest). In addition, these data indicated that although GM-CSF and IFN-γ production are regulated differently during infection, these pathways converge as the immune response to *M. tuberculosis* matures and significant numbers of dual cytokine-producing T cells begin to accumulate in the lung.

### GM-CSF production by T cells can mediate protection *in vivo.*

**FIG 4:**
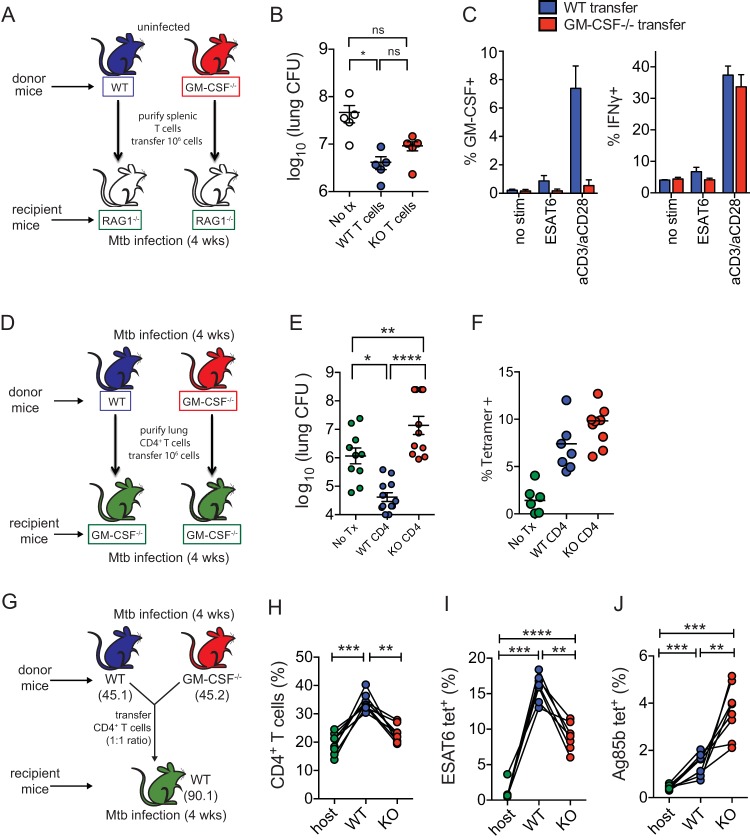
T cell-derived GM-CSF controls *M. tuberculosis* growth. (A) Experimental strategy for transfer of donor WT or GM-CSF^−/−^ T cells into RAG^−/−^ recipient mice, followed by *M. tuberculosis* aerosol infection. (B) CFU in lungs 4 weeks postinfection after WT or GM-CSF^−/−^ T cells were transferred into RAG^−/−^ recipients. Data are representative of five independent experiments. (C) Percentage of CD4^+^ T cells producing IFN-γ or GM-CSF after 5 h of ESAT6_1-20_ peptide stimulation or anti-CD3/anti-CD28 stimulation with brefeldin A and IL-2 at 4 weeks postinfection. (D) Experimental strategy for adoptive transfer experiments using sublethally irradiated GM-CSF^−/−^ mice as recipients, who were then infected with *M. tuberculosis* via aerosol. (E) CFU in lungs 4 weeks postinfection after WT or GM-CSF^−/−^ CD4^+^ T cells were transferred into GM-CSF^−/−^ recipients. Data were compiled from 4 independent infections with a total of *n* = 16 to 19 mice per condition. (F) Frequency of ESAT6_3-17_ tetramer-positive CD4^+^ T cells from donor WT and GM-CSF^−/−^ infected mice 4 weeks after sublethal irradiation, adoptive transfer, and aerosol infection. (G) Experimental strategy for adoptive cotransfer of CD4^+^ T cells from donor WT and GM-CSF^−/−^ Thy1.1 recipients 4 weeks after infection (at a 1:1 ratio) with *M. tuberculosis* infection via aerosol. (H) Origin of CD4^+^ T cells based on congenic markers 4 weeks after infection. (I and J) Frequency of ESAT6_3-17_-positive (I) or Ag85b_241-256_ tetramer-positive (J) CD4^+^ T cells among total host (e.g., endogenous), WT, or KO CD4^+^ T cells. Statistical testing was performed by using a paired 1-way ANOVA. *, *P* < 0.05; **, *P* < 0.01; ****, *P* < 0.0001. Error bars indicate SEM.

To determine the relative importance of GM-CSF production by T cells for control of *M. tuberculosis* infection, we used two different adoptive transfer strategies. First, bulk T cells from uninfected WT or GM-CSF^−/−^ mice were transferred into RAG^−/−^ mice, which were then infected with a low dose of aerosolized *M. tuberculosis* (Fig. 4A). Both WT and GM-CSF^−/−^ CD4^+^ T cells transferred similar amounts of protection to immunodeficient RAG^−/−^ mice (Fig. 4B), and we confirmed, by intracellular cytokine staining, that WT and GM-CSF^−/−^ CD4^+^ T cells were both able to produce IFN-γ after transfer in response to either antigen or anti-CD3/CD28 stimulation, independently of GM-CSF production (Fig. 4C). In some experiments, WT CD4^+^ T cells were more efficient than GM-CSF^−/−^ CD4^+^ T cells at transferring protection; however, these differences were not always statistically significant. An important caveat to this approach is that in this model recipient mice still have an intact non-T cell source of GM-CSF, including lung epithelial cells, analogous to radioresistant cells. Indeed, both we (Fig. 2) and others (27, 28) have shown that such cells are crucial for protection in the absence of T cell production of GM-CSF. Therefore, we tested a second model in which the only source of GM-CSF was the transferred T cells.

To study the protective capacity of T cell-derived GM-CSF, we transferred WT or GM-CSF^−/−^ CD4^+^ effector T cells, obtained from *M. tuberculosis*-infected WT or GM-CSF^−/−^ mice, into sublethally irradiated GM-CSF^−/−^ mice (Fig. 4D). In this experiment, we transferred lung-derived CD4^+^ T cells from previously infected mice in order to transfer the greatest number of *M. tuberculosis* antigen-specific T cells possible, since their population is significantly expanded in infected mice compared to naive mice. This adoptive transfer of immune T cells was adapted from previously published adoptive transfer strategies (46, 47). GM-CSF^−/−^ effector CD4^+^ T cells transferred to GM-CSF^−/−^ mice did not confer protection in the lung or the spleen (Fig. 4E and data not shown). In fact, they exacerbated disease and growth of *M. tuberculosis*. In contrast, WT effector CD4^+^ T cells transferred to GM-CSF^−/−^ mice conferred significant protection: there was an ~1.7 Δlog_10_ decreased bacterial burden compared to GM-CSF^−/−^ mice that did not receive T cell transfer and ~2.5 Δlog_10_ decreased burden compared to mice that received GM-CSF^−/−^ effector CD4^+^ T cells (Fig. 4E). The inability of the GM-CSF^−/−^ effector CD4^+^ T cells to transfer protection led us to verify the quality of the immune response in the donor mice. We measured the frequency of ESAT6_3-17_-specific CD4^+^ T cells among the purified CD4^+^ T cells by using tetramers. We found that transferred CD4^+^ T cells from both donor WT and GM-CSF^−/−^ infected mice had similar frequencies of ESAT6_3-17_-specific CD4^+^ T cells (2.0% to 2.5% of the CD4^+^ T cells). Similarly, at the 4-week time point, the median frequency of ESAT6_3-17_-specific CD4^+^ T cells was slightly higher among recipients that received GM-CSF^−/−^ CD4^+^ T cells than mice that received WT CD4^+^ T cells (9.8% versus 7.4%), possibly reflecting the higher lung bacterial burden in the former group, but the difference was not statistically different between the subjects that received WT versus GM-CSF^−/−^ CD4^+^ T cells (Fig. 4F).

To further verify that the GM-CSF^−/−^ CD4^+^ T cells were fit and could expand following *M. tuberculosis* challenge, we cotransferred congenically marked WT (CD45.1) or GM-CSF^−/−^ (CD45.2) CD4^+^ effector T cells obtained from *M. tuberculosis*-infected WT or GM-CSF^−/−^ mice into sublethally irradiated CD90.1 recipient mice. After transfer, the mice were infected, and we measured the relative fitness of the T cells 4 weeks later (Fig. 4G). We were able to identify recipient, WT donor, and GM-CSF^−/−^ donor CD4^+^ T cells (Fig. 4H). The WT CD4^+^ T cells were more abundant than GM-CSF^−/−^ CD4^+^ T cells, with a frequency of 34% versus 23% (*P* < 0.0004, paired *t* test) (Fig. 4H). However, the relative frequency of antigen-specific CD4^+^ T cells derived from each genotype differed depending on the epitope: WT CD4^+^ T cells dominated the response to ESAT6, while GM-CSF^−/−^ CD4^+^ T cells more frequently recognized Ag85b (Fig. 4I and J). The results of the cotransfer experiment suggest that GM-CSF^−/−^ T cells do not have a broad defect in their ability to expand and generate antigen-specific responses that would explain their failure to control infection when transferred.

Taken together, these different adoptive transfer experiments indicate that while GM-CSF is not required for T cell-mediated protection in a setting where GM-CSF is produced by other cell types, T cell production of GM-CSF can be crucial for the control of *M. tuberculosis* growth in environments where nonhematopoietic GM-CSF production is suboptimal.

### GM-CSF production by human T cells.

**FIG 5:**
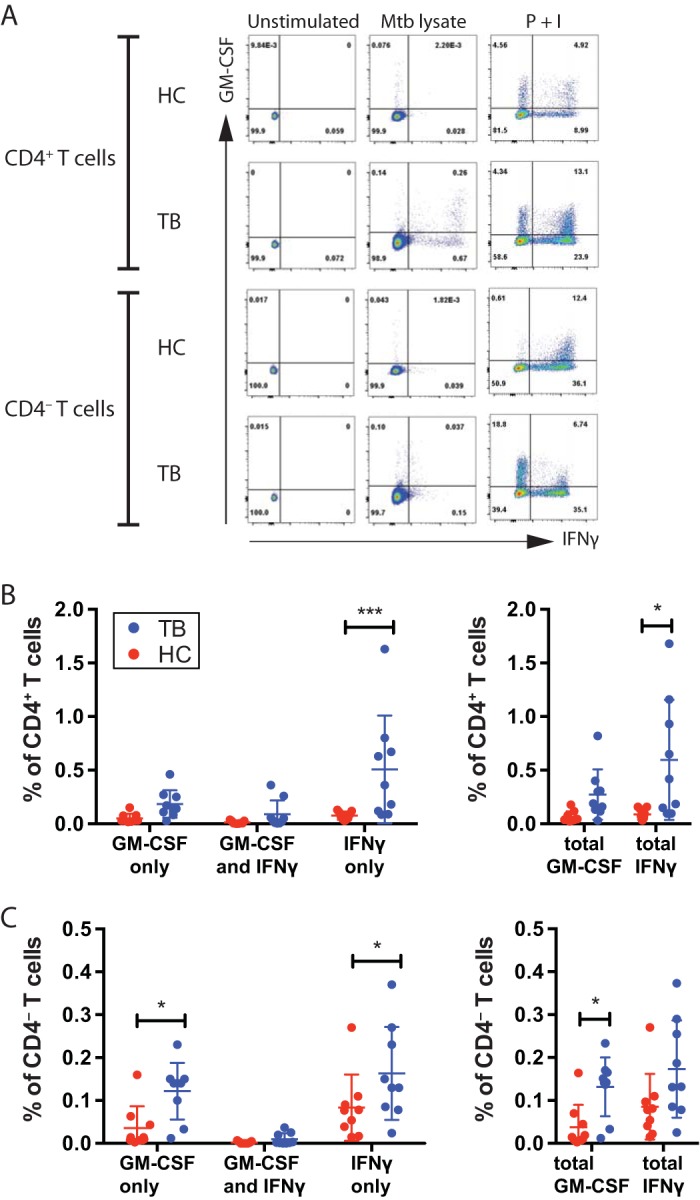
GM-CSF production by human T cells. (A) Representative flow cytometric analysis results for GM-CSF and IFN-γ production by unstimulated human peripheral blood CD4^+^ and CD4^−^ T cells, or after stimulation with *M. tuberculosis* lysate (Mtb lysate), or after treatment with PMA plus ionomycin (P + I). (B and C) The frequency of CD4^+^ (B) or CD4^−^ (C) T cells producing GM-CSF only, both GM-CSF and IFN-γ, or IFN-γ only (left), or the total GM-CSF or total IFN-γ (right), from healthy controls (*n* = 9) or active TB patients (*n* = 9) stimulated with *M. tuberculosis* lysate. Red circles, HC; blue circles, TB patients. Lines indicate means ± standard deviations. Statistical testing was performed with a two-way ANOVA. *, *P* < 0.05; **, *P* < 0.01; ****, *P* < 0.0001.

To investigate whether human T cells produce GM-CSF during infection, peripheral blood mononuclear cells (PBMC) from active TB patients (TB) or healthy controls (HC) were stimulated or not with *M. tuberculosis* lysate, and the production levels of GM-CSF and IFN-γ by CD4^+^ T cells and CD4^−^ T cells were determined by flow cytometry (see Fig. S1 in the supplemental material for the gating strategy). CD4^−^ T cells consist primarily of classical CD8^+^ T cells but also include CD4^−^ CD8^−^ T cells, such as iNKT cells and γδ T cells. GM-CSF was detected in stimulated CD4^+^ and CD4^−^ T cells from HC and TB subjects, compared to an isotype control (data not shown) or under unstimulated conditions (Fig. 5A). In addition, stimulation with phorbol myristate acetate (PMA) and ionomycin led to a substantial proportion of CD4^+^ T cells producing GM-CSF, either alone or in combination with IFN-γ. CD4^−^ T cells also produced GM-CSF under these stimulation conditions (Fig. 5A). We next determined whether GM-CSF production discriminated between HC and TB subjects (Fig. 5B). Although stimulation with *M. tuberculosis* lysate led to an increase in GM-CSF production by CD4^+^ T cells from TB patients, GM-CSF production was not significantly elevated compared to HC subjects. In contrast, CD4^+^ T cell production of IFN-γ was significantly increased in TB patients compared to HC, both for CD4^+^ T cells that only produced IFN-γ (Fig. 5B, left) as well as the total percentage of IFN-γ-producing CD4^+^ T cells, with or without GM-CSF (Fig. 5B, right), as anticipated. Somewhat unexpectedly, the percentage of CD4^−^ T cells producing GM-CSF after stimulation with the *M. tuberculosis* lysate was significantly increased in TB patients compared to HC subjects both for CD4^−^ T cells that only produced GM-CSF (Fig. 5C, left) and for the total percentage of GM-CSF-producing CD4^−^ T cells (Fig. 5C, right). These data show that both CD4^+^ and CD4^−^ T cells produce GM-CSF as part of the human T cell response to *M. tuberculosis* and that inclusion of GM-CSF in immunoassays may increase their predictive power (48).

10.1128/mBio.01514-17.1 FIG S1  Gating strategy for human PBMC. Lymphocytes were identified based on typical FSC and SSC patterns, and then singlets were gated to avoid cell clumps. CD4^+^ T cells were identified based on their dual staining with antibodies specific for CD3 and CD4, and CD4^−^ T cells were defined by positive staining for CD3 and negative staining for CD4. Download FIG S1, PDF file, 0.3 MB. Copyright © 2017 Rothchild et al. 2017 Rothchild et al. This content is distributed under the terms of the Creative Commons Attribution 4.0 International license.

### GM-CSF and IFN-γ have an additive effect in promoting macrophage control of intracellular bacterial replication.

**FIG 6:**
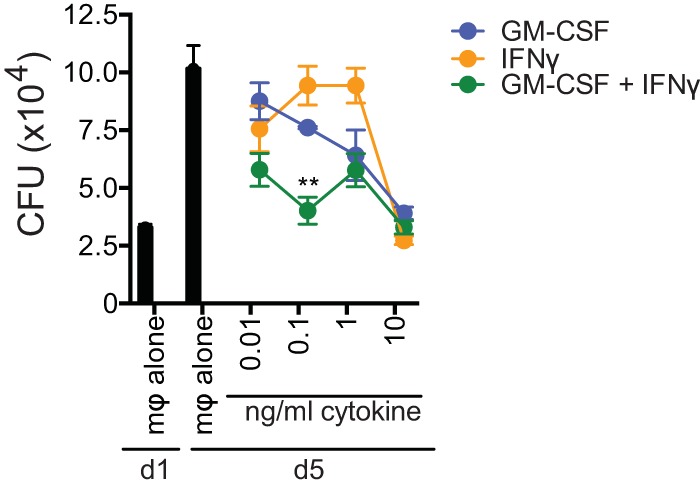
GM-CSF and IFN-γ have an additive antimicrobial effect. Mycobacterial growth inhibition was performed using H37Rv-infected C57BL/6 macrophages, which were infected overnight as described elsewhere (23). CFU were measured on day 1 (macrophages [mφ] alone, baseline) and on day 5 postinfection. Recombinant GM-CSF and/or IFN-γ was added on day 1 postinfection. Error bars indicate means ± SEM. **, *P* < 0.01. One-way ANOVA with Dunnett’s posttest was used to compare combination treatment with individual cytokine treatment at each concentration. Data are representative of two independent experiments.

GM-CSF activates macrophages to limit intracellular growth of *M. avium* and *M. tuberculosis* (24–26). The finding that a significant proportion of CD4^+^ T cells produced both GM-CSF and IFN-γ, in both mice and humans, led us to hypothesize that GM-CSF and IFN-γ work together to activate macrophages to limit intracellular bacterial growth. Using an *in vitro* macrophage infection model (23), we tested whether the antimicrobial effects of GM-CSF and IFN-γ were additive (Fig. 6). Both GM-CSF and IFN-γ were individually able to inhibit *M. tuberculosis* growth at higher cytokine concentrations (such as 10 ng/ml). However, at lower concentrations (0.1 ng/ml), the combination of GM-CSF and IFN-γ led to a statistically significant decrease in CFU compared to the efficacy of each cytokine alone. The lower cytokine concentrations may be more representative of the concentrations encountered by infected cells in the lung, and under these conditions, GM-CSF and IFN-γ appear to have an additive effect. Although the results do not reveal a clear synergistic effect between these cytokines, further investigation into the antimicrobial mechanism of GM-CSF may reveal mechanistic connections with IFN-γ.

### GM-CSF antimicrobial activity requires PPARγ expression in macrophages.

To identify the antimicrobial mechanism of GM-CSF, we first tested a number of pathways previously reported to be modulated by GM-CSF (49–51). Treatment of macrophages with recombinant GM-CSF did not induce nitric oxide or reactive oxygen species (ROS) production or increase the rate of phagocytosis (data not shown). To examine the effect of GM-CSF more broadly, we focused our investigation on transcription factors that might be required for GM-CSF-mediated control of *M. tuberculosis* growth within macrophages. PPARγ is a nuclear receptor that regulates cellular lipid metabolism and plays an important role in lipid recycling and surfactant catabolism, especially in alveolar macrophages (43, 52–54). Furthermore, PPARγ has specifically been linked to GM-CSF function based on the finding that patients with (e.g., PAP) often have autoantibodies to GM-CSF or mutations in the receptor for GM-CSF, which leads to dysregulated surfactant metabolism by alveolar macrophages. Importantly, the PAP-like phenotype of GM-CSF^−/−^ animals is rescued by lentivirus-directed overexpression of PPARγ, leading to net cholesterol efflux and reduction of lipid accumulation in alveolar macrophages (55). Therefore, we hypothesized that PPARγ mediates the protective effects of GM-CSF in macrophages.

**FIG 7:**
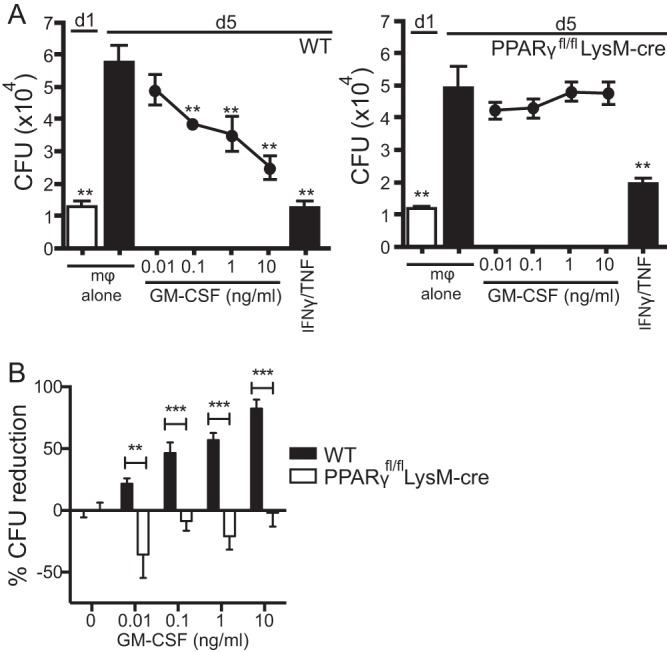
GM-CSF antimicrobial activity requires PPARγ expression in macrophages. (A) Mycobacterial growth inhibition was performed using H37Rv-infected macrophages from C57BL/6 mice (e.g., WT) (left) or PPARγ^fl/fl^; LysM-cre mice (right). Baseline CFU were measured on day 1 in the absence of GM-CSF. Recombinant cytokines, including GM-CSF (0.01 to 10 ng/ml), were added on day 1, and bacterial growth was determined on day 5. Recombinant IFN-γ (10 U/ml) and TNF (10 ng/ml) were used as positive controls. (B) The percent CFU reduction compiled from 4 independent experiments. Error bars indicate means ± SEM. **, *P* < 0.01; ***, *P* < 0.001. One-way ANOVA with Dunnett’s posttest was used to compare day 5 untreated macrophages for the data in panel A; multiple Student’s *t* tests were used for the data in panel B. Data are representative of four independent experiments with three replicates each. mφ, macrophages.

H37Rv-infected WT and PPARγ^−/−^ (PPARγ^fl/fl^; LysM-cre) peritoneal macrophages were treated with recombinant GM-CSF. While GM-CSF led to inhibition of bacterial growth in WT macrophages, no inhibition was observed for PPARγ^−/−^ macrophages (Fig. 7A). IFN-γ/TNF treatment, a positive control for bacterial growth restriction, led to inhibition of bacterial growth in both macrophages types. When the percent CFU reduction was calculated for 4 independent experiments, there was a statistically significant difference for the CFU reduction between WT and PPARγ^−/−^ macrophages for all GM-CSF doses between 0.01 and 10 ng/ml (Fig. 7B). Similar results were found for comparisons between PPARγ^−/−^ (PPARγ^fl/fl^; LysM-cre) and PPARγ^fl/fl^ control (PPARγ^fl/fl^; no cre expression) macrophages (data not shown). These data suggest that PPARγ signaling is involved in the antimicrobial effector pathway stimulated by GM-CSF in *M. tuberculosis*-infected macrophages.

## DISCUSSION

While it is well-understood that T cells, particularly CD4^+^ T cells, are required to control *M. tuberculosis* infection, the particular T cell effector functions that lead to control of infection continue to be debated. Indeed, the paradigm that CD4^+^ T cell production of IFN-γ is the crucial link between T cell immunity and control of TB has been broken (56). Although IFN-γ is crucial for host resistance and CD4^+^ T cell production of IFN-γ can be essential (10), it is clear that IFN-γ-independent mechanisms of host protection exist and IFN-γ production by CD4^+^ T cells is not sufficient on its own for protection (11, 12, 57). Furthermore, at least some of the host-protective nature of IFN-γ may be based on an immunoregulatory role (57). Regardless, it is increasingly appreciated that IFN-γ is a better correlate of bacterial load than protection (58) and that, under certain circumstances, too much IFN-γ can be detrimental (13). Given the rapidly changing views on the role of IFN-γ, what T cell effector molecules activate macrophages to restrict intracellular *M. tuberculosis* growth?

We previously made the unexpected observation that iNKT cells control intracellular *M. tuberculosis* growth (59) by an IFN-γ-independent mechanism (23). By taking advantage of our *in vitro* system, we identified GM-CSF as an iNKT cell product that activates macrophages to control infection (23). Indeed, this finding verified previous reports that GM-CSF has antimycobacterial activity, as originally described by Denis et al. (26). In addition, GM-CSF expression adenovirus therapy was recently shown to have some efficacy against both drug-sensitive and drug-resistant *M. tuberculosis* infection in mice (41). Furthermore, the GM-CSF^−/−^ mouse is highly susceptible to *M. tuberculosis* infection, with a median survival time of 4 to 5 weeks (27, 28). However, the role of GM-CSF is complicated, as it has pleiotropic effects with a particularly instrumental role in alveolar macrophage development in the lung (60), although surprisingly, other myeloid cell populations in the GM-CSF^−/−^ mouse are relatively normal (36). A key function for GM-CSF in lung physiology is its role in surfactant recycling, and the GM-CSF^−/−^ mouse develops a pulmonary syndrome that resembles the human disease PAP, which has been associated with the production of anti-GM-CSF antibodies. Therefore, it has been uncertain whether the susceptibility of the GM-CSF^−/−^ mouse is due to the lack of mature alveolar macrophages, abnormal surfactant metabolism, or some role of GM-CSF in adaptive immunity. The finding by Higgins et al., which was confirmed herein, that lung GM-CSF increases during active tuberculosis raises the possibility that GM-CSF is produced as part of the acquired immune response during *M. tuberculosis* infection (45). Indeed, the kinetics of GM-CSF accumulation in the lung largely mirrors the kinetics of T cell recruitment following priming.

Our finding that iNKT cell production of GM-CSF contributes to antimycobacterial immunity in an *in vitro* model with *M. tuberculosis*-infected macrophages supports this concept (23). We also detected that iNKT cells in the lungs of *M. tuberculosis*-infected mice produced GM-CSF in a CD1d-restricted manner. However, since CD1d-restricted T cells are not required for host resistance against *M. tuberculosis* infection in the mouse model (61, 62), we wondered whether the production of GM-CSF by multiple T cell subsets might make an important contribution to protection against *M. tuberculosis* infection. Expression of GM-CSF by type II pulmonary epithelial cells partially corrected the susceptibility of GM-CSF^−/−^ mice (27, 28). Indeed, our result showing that GM-CSF production by radioresistant cells (e.g., nonhematopoietic cells) was sufficient to confer host resistance is consistent with these previous reports. However, we also found that GM-CSF production by radiosensitive cells was capable of conferring host resistance. In fact, we showed that different types of T cells produce GM-CSF early after infection, most prominently γδ T cells and iNKT cells. As immunity to *M. tuberculosis* becomes established, GM-CSF production by CD4^+^ T cells dominates.

We next asked whether GM-CSF production by effector T cells is important for T cell-mediated protection against *M. tuberculosis*. The straightforward approach of transferring naive splenic T cells from WT or GM-CSF^−/−^ mice into RAG^−/−^ mice gave us somewhat inconclusive results. In general, both populations of T cells protected RAG^−/−^ mice, and in some experiments, WT T cells were more effective. These transfer experiments suggest that GM-CSF is not required for T cell-mediated protection. However, we realized that these experiments might be confounded by the fact that GM-CSF production by radioresistant cells can mask the effect of GM-CSF produced by T cells. Because of these concerns, we tested a second model. We infected WT or GM-CSF^−/−^ mice to first generate effector T cells. CD4^+^ T cells were then purified from the lungs of these mice at around 4 weeks of infection, near the peak of the T cell response. These T cells were then adoptively transferred into GM-CSF^−/−^ mice, which were then challenged with *M. tuberculosis*. Under these conditions, WT CD4^+^ T cells transferred protection, and this protection was significantly better than that after transfer of GM-CSF^−/−^ CD4^+^ T cells. Surprisingly, transfer of GM-CSF^−/−^ CD4^+^ T cells generally led to increased bacterial growth. Although our experiments were performed in sublethally irradiated mice, T cells transferred into unirradiated GM-CSF^−/−^ recipient mice produced the same results. One interpretation is that GM-CSF directly induces antimicrobial activity in macrophages (as we observed for iNKT cells). Alternatively, GM-CSF may be important in T cell priming, differentiation, and expansion into antigen-specific effector T cells. To test these various interpretations, we cotransferred WT and GM-CSF^−/−^ CD4^+^ T cells from *M. tuberculosis*-infected mice into sublethally irradiated recipients and monitored the ability of the T cell population to expand during infection. While the GM-CSF^−/−^ CD4^+^ T cells expanded to a slightly lower frequency overall than the WT CD4^+^ T cells after 4 weeks, their ability to expand in an antigen-specific manner was highly dependent on the antigen. ESAT6-specific GM-CSF^−/−^ CD4^+^ T cells were observed at a lower frequency than their WT counterparts, but Ag85b-specific GM-CSF^−/−^ CD4^+^ T cells were found at a higher frequency than WT cells. This result is intriguing, because a recent study showed that in both mice and humans the functions of ESAT6 and Ag85b CD4^+^ T cells are highly distinct and a direct result of antigen availability over the course of infection (63). Our results do not eliminate the possibility that GM-CSF production by T cells have both direct effects to activate macrophages to kill *M. tuberculosis* and indirect (i.e., regulatory) effects that promote host resistance.

We interpret these various data to mean that in a GM-CSF-replete environment, for example, a WT mouse with a functioning epithelial compartment, T cell production of GM-CSF is not absolutely required for protection. However, if there is a deficit in local GM-CSF production, because of disrupted epithelium, for example, T cell production of GM-CSF can become crucial for bacterial control. In most hosts, both IFN-γ and GM-CSF will be present in the lung, whether made by the same cell type (e.g., T cells) or different cells (e.g., T cells and epithelial cells). Therefore, we tested whether the antibacterial activity that IFN-γ and GM-CSF stimulate in macrophages was additive. In general, we found that at lower concentrations of cytokines, IFN-γ and GM-CSF had an additive effect that is possibly synergistic. This is important, as it implies that distinct molecular pathways are activated by these two cytokines.

It was notable to us that in the reciprocal radiation chimeric mouse experiments, the presence of GM-CSF-producing cells had an effect on the bacterial burden in the lung, but not in the spleen. This suggested that GM-CSF has a protective function during *M. tuberculosis* infection that is lung specific. Based on the established link between GM-CSF and PPARγ in patients with PAP and in GM-CSF^−/−^ mice (38, 55, 64, 65), the link between anti-GM-CSF antibodies and mycobacterial infection (40), and the role of PPARγ in innate immunity to mycobacteria (66–68), we considered whether the macrophage effector function of GM-CSF is associated with PPARγ expression. In our model, the antibacterial action of GM-CSF is absolutely dependent upon PPARγ expression. GM-CSF has a variety of actions on different cell types, including phagocytosis stimulation, lipid body formation, and nitric oxide (NO) induction. Sorting out which of these functions contributes to intracellular control of *M. tuberculosis* growth is difficult, since many of these cellular processes are known to affect bacterial survival. While some of these functions, like phagocytosis, NO, and ROS production, were not increased in primary peritoneal macrophages after GM-CSF treatment, other functions, like lipid body formation and cell growth and survival, were increased after GM-CSF treatment but were not dependent on PPARγ. This allowed us to rule out many of the effector functions previously attributed to GM-CSF, although we have not yet pinpointed the pathway(s) downstream of PPARγ that is critical for GM-CSF action. Although the consequences of GM-CSF signaling on PPARγ could vary based on macrophage subtype and baseline expression of PPARγ (69), the effect of GM-CSF deficiency on PPARγ-regulated genes is similar in alveolar and peritoneal macrophages (70). Characterization of this pathway has been made more difficult by the fact that previous studies have shown that PPARγ plays a critical role in the development of alveolar macrophages in the lung. LysM-cre/PPARγ^fl/fl^ mice have relatively normal numbers of alveolar macrophages but go on to develop a mild form of pulmonary alveolar proteinosis as they age (>4 months) (38,71). A shortcoming of our study is that we used thioglycolate-elicited peritoneal macrophages instead of alveolar macrophages. The large number of macrophages needed for the CFU assay makes it impractical to use alveolar macrophages.

The strongest evidence that GM-CSF is essential for host defense against infection in people comes from clinical observations linking the presence of anti-GM-CSF autoantibodies with susceptibility to cryptococcal meningitis and pulmonary TB (40). We hypothesized that GM-CSF production might be part of the human T cell response against mycobacteria. In a pilot study, we evaluated whether differences existed in the capacity of T cells from healthy subjects that were free of TB disease versus the capacities of the T cells in patients diagnosed with pulmonary TB to produce GM-CSF. Both CD4^+^ and CD4^−^ T cells from subjects with active disease produced GM-CSF after stimulation with *M. tuberculosis* lysate. Some T cells produced only GM-CSF and others produced GM-CSF and IFN-γ, suggesting that they may represent two distinct T cell subsets (72). Interestingly, GM-CSF production by CD4^−^ T cells was greater in TB patients than in HC. While the specific human CD4^−^ T cell subsets that produce GM-CSF in response to *M. tuberculosis* (e.g., classical CD8^+^ T cells, iNKT cells, γδ T cells) could not be identified here, these results showed that GM-CSF is produced by T cells as part of the immune response to *M. tuberculosis* in people. A potential confounder with the study is that the healthy subjects were presumed to be vaccinated with BCG, and T cells elicited by BCG may have also recognized antigens present in the *M. tuberculosis* lysates. We hypothesize that GM-CSF has a role in controlling infection, and hence it could be predicted to be more abundant in subjects that are latently infected. Another possibility is that GM-CSF-producing T cells are more abundant during the early stages of infection, as part of an initial Th17 response (73), and skewing toward a Th1 response may lead to the loss of GM-CSF production by CD4^+^ T cells (74). Relatively little is understood about the regulation of GM-CSF production by T cells. There does not appear to be a master regulator that programs GM-CSF production, although recently a subset of T cells was described in which GM-CSF was the dominant cytokine (72). Importantly, although GM-CSF is associated with Th17 responses in mice, it appears to be associated with Th1 responses in humans (75). Nevertheless, GM-CSF is an important cytokine made by T cells, and it has a crucial role in the pathogenicity of autoreactive Th1 or Th17 cell during autoimmune neurological disease. We hypothesize that generation of *M. tuberculosis*-specific T cells that can make GM-CSF may enhance protection against *M. tuberculosis* infection. We envision a role for GM-CSF either in host-directed therapy or possibly in the development of vaccines that elicit GM-CSF-producing T cells.

## MATERIALS AND METHODS

### Ethics statement.

All animal experiments were performed in accordance with guidelines of the Office of Laboratory Animal Welfare and the Public Health Service Policy on Humane Care and Use of Laboratory Animals. The protocols that adhered to these guidelines were approved by the Dana Farber Cancer Institute Animal Care and Use Committee (Animal Welfare Assurance number A3023-01) or by the Department of Animal Medicine of the University of Massachusetts Animal Care and Use Committee (Animal Welfare Assurance number A2420-13). All mice were bred and maintained using standard humane animal husbandry protocols. Mice infected with *M. tuberculosis* were housed in a biosafety level 3 facility under specific-pathogen-free conditions in animal biohazard containment suites (Dana Farber Cancer Institute, Boston, MA, and University of Massachusetts, Worcester, MA). Collection of the human blood samples was approved by the Institutional Review Board of Shenzhen Third People’s Hospital (ethical approval number 2016-006), and the methods were performed in accordance with the approved guidelines of the institution. All subjects were adults, and written informed consent was obtained from all participants.

### Mice.

C57BL/6J (WT) and B6.129S7-Rag1/J (RAG^−/−^) mice were obtained from Jackson Laboratories. C57BL/6J GM-CSF^−/−^ mice were provided by Glenn Dranoff. C57BL/6J PPARγ^fl/fl^;LysMcre^+/+^ (PPARγ^−/−^) and PPARγ^fl/fl^; LysMcre^−/−^ (PPARγ^fl/fl^ control) mice were provided by Glenn Dranoff.

### Reciprocal bone marrow chimeras.

Bone marrow chimeras were made by lethally irradiating CD45.2 GM-CSF^−/−^ mice and C57BL/6J WT recipients (2 doses of 600 rad separated by 3 h). BM was flushed from the femurs, tibia, and humeri of donor mice (CD45.1 GM-CSF^−/−^ mice and C57BL/6J WT mice), and red blood cells were lysed. Each recipient mouse received a total of 10^7^ BM cells via lateral tail vein injection and was kept on antibiotic-containing water for 5 weeks following irradiation. Mice were checked for reconstitution to assess the ratio of donor cells in the peripheral blood by flow cytometry. BM chimeras were infected with *M. tuberculosis* 8 to 10 weeks after transfer of the BM cells (76).

### *In vivo* aerosol infections.

*In vivo* infections were performed using virulent *M. tuberculosis* (Erdman strain). For each infection, a bacterial aliquot was thawed, sonicated twice for 10 s, and then diluted in 0.9% NaCl–0.02% Tween 80. A 15-ml suspension of *M. tuberculosis* was loaded into a nebulizer (MiniHEART nebulizer; Vortran Medical Technology); mice were infected via the aerosol route with a nose-only exposure unit (Intox Products) and received ~50 to 100 CFU/mouse. Alternatively, mice were infected using a Glas-Col inhalation exposure system (Terre Haute, IN). Mice were euthanized by CO_2_ inhalation, and lungs were aseptically removed after perfusion of 10 ml of sterile RPMI medium into the right ventricle of the heart. Lung mononuclear cells were obtained by mechanical disruption using a gentleMACS dissociator (Miltenyi Biotec, Inc.) followed by incubation in collagenase (Sigma-Aldrich) for 30 min at 37°C. Cells were isolated by forcing suspensions through a 70-μm cell strainer and then enumerated in 4% trypan blue by using a hemacytometer.

### T cell adoptive transfer.

For bulk T cell transfers, naive T cells were isolated from the spleen and lymph nodes of WT and GM-CSF^−/−^ mice and then separated using the pan T cell isolation kit (Miltenyi Biotec, Inc.) following the manufacturer’s protocol. A total of 5 × 10^6^ T cells were then injected intravenously (i.v.) via tail vein into each RAG^−/−^ recipient. All mice were infected with *M. tuberculosis* Erdman strain via the aerosol route within 24 h of cell transfer. For CD4^+^ T cell cotransfer experiments, immune CD4^+^ T cells from the lungs of WT (CD45.1) and GM-CSF KO (CD45.2) mice infected by aerosol with *M. tuberculosis* Erdman strain for 4 weeks were purified by positive separation with anti-CD4 magnetic beads using an autoMACS Pro separator (Miltenyi Biotec, Inc.). Cell purity was consistently ≥95% for WT CD4^+^ T cells and ≥85% for GM-CSF KO CD4^+^ T cells. CD4^+^ cells (10^6^ of each genotype) were injected i.v. into recipient GM-CSF^−/−^ mice that had been sublethally irradiated 24 h before with 600 rad from a cesium 137 source. Within 24 h of cell transfer, recipient mice were infected with aerosolized *M. tuberculosis* Erdman. For cotransfer experiments, immune CD4^+^ T cells from the lungs of WT (CD45.1) and GM-CSF KO (CD45.2) mice were cotransferred (10^6^ each) into sublethally irradiated WT CD90.1 recipients, which were then infected with aerosolized *M. tuberculosis* Erdman, following the protocol described above.

### Flow cytometry and ICS.

For ICS stimulation, cells were plated in a 96-well plate and incubated for 4 to 5 h at 37°C with IL-2 in either the absence of stimuli or in the presence of TB10.4_4-11_ peptide (10 μM; New England Peptide), ESAT6_1-20_ peptide (10 μM; New England Peptide), or anti-CD3/anti-CD28 (1 µg/ml; BioLegend). Brefeldin A (GolgiPlug; BD Biosciences) was added to the cultures 1 h after the addition of exogenous stimuli. Cells were next incubated with CD16/CD32 (FcBlock; BD Biosciences). Surface staining included antibodies for murine TCR-β (H57-597), TCR-γδ (UC7-13D5), CD3 (17A2), CD19 (6D5), CD4 (RM4-5), CD8 (53-6.7), CD25 (PC61), CD69 (H1.2F3), CD11b (M1/70), CD11c (N418), Ly6C (HK1.4), Ly6G (1A8), and isotype controls (all from BioLegend). Except for iNKT cell staining, ICS with antibodies specific for mouse GM-CSF (MP1-22E9; EBioscience) and IFN-γ (XMG1.2; BioLegend) was performed in Perm/Wash buffer (BD Biosciences) following fixation and permeabilization with Fix/Perm buffer (BD Biosciences). PBS-57-loaded and control phycoerythrin- and allophycocyanin-conjugated CD1d tetramers were provided by the National Institute of Allergy and Infectious Diseases Tetramer Facility (Emory University Vaccine Center). For iNKT cells, after tetramer staining, ICS with antibodies specific for mouse GM-CSF and IFN-γ (see above) was performed following fixation with 4% paraformaldehyde and permeabilization with Perm/Wash buffer (BD Biosciences). Data were collected using FACSCanto (BD Biosciences) or MACSQuant (Miltenyi Biotec, Inc.) systems and analyzed with FlowJo software (Tree Star, Inc.).

### Human TB patients and healthy controls.

Healthy adults (*n* = 9) and patients with TB (*n* = 9) were recruited from Shenzhen Third People’s Hospital. For all subjects, a medical history was taken and a physical examination was performed with routine clinical investigations, including HIV serology, chest radiography, IFN-γ release assays, and microbiological sputum examination, whenever possible. Patients with HIV infection were excluded. Case definitions were as follows: (i) HC had a negative chest X-ray, no evidence of TB, no history of TB, and negative results for an *M. tuberculosis*-specific IFN-γ enzyme-linked immunosorbent spot (ELISPOT) assay (77). (ii) All recruited TB patients had symptoms and chest computed tomography results suggestive of pulmonary TB, as well as a positive sputum *M. tuberculosis* culture. The mean age of each group (with interquartile range) was as follows: HC, 44 years (25 to 79); TB, 40 years (25 to 68). The male:female ratio of each group was 5:4 for HC and 5:4 for the TB group.

### Human cell preparation and *in vitro* stimulation.

PBMC were isolated from whole blood as described previously (78). PBMC were cultured in complete RPMI 1640 medium at a final concentration of 10^6^/ml with PMA and ionomycin (50 ng/ml and 1 mg/ml; Sigma-Aldrich, USA) for 2 h in a 24-well plate at 37°C in 5% CO_2_. Brefeldin A (10 mg/ml; Sigma-Aldrich) was then added and the incubation was continued for an additional 4 h. To measure antigen-specific cytokine production, heat-killed *M. tuberculosis* lysate (10 mg/ml) was added to the PBMC for 4 h, and then brefeldin A was added and the cells were cultured for an additional 12 h. PBMC incubated with no stimulation served as negative controls. After 16 h of culture, cells were stained with surface and intracellular cytokine antibodies for flow cytometric analysis as described below. Monoclonal antibodies against human IFN-γ (4S.B3) and GM-CSF (BVD2-21C11) were obtained from BioLegend. All other reagents were obtained from BD Biosciences, including monoclonal antibodies against human CD3 (SK7), CD4 (SK3), and isotype-matched control immunoglobulins. After stimulation, cells were washed twice with phosphate-buffered saline (PBS) and fixed in BD fluorescence-activated cell sorting (FACS) lysing solution followed by permeabilization using FACS permeabilizing solution and then stained for the intracellular cytokines and molecules with antibodies diluted in PBS buffer. Lymphocytes were gated on forward- and side-scatter profiles. At least 0.2 million events were acquired using a FACSCanto system (Becton, Dickinson, San Jose, CA, USA) and analyzed using FACSDiva software (version 5.0.2).

### Macrophage isolation and culture.

Thioglycolate-elicited peritoneal macrophages were lavaged 4 to 5 days after 3% intraperitoneal thioglycolate injection and then isolated by positive selection with CD11b microbeads and LS magnetic columns (Miltenyi Biotec, Inc.). Purified cells were over 95% F4/80^+^ CD11b^+^, as determined by flow cytometry. Macrophages were seeded at 1 × 10^5^ in 96-well culture plates in complete RPMI 1640 medium (Invitrogen Life Technologies, Inc.) supplemented with 10% fetal calf serum (HyClone).

### *M. tuberculosis in vitro* culture and infection.

H37Rv was grown and prepared as previously described (59). Bacteria were counted and added to macrophages at an effective multiplicity of infection (MOI) of 0.2 for CFU experiments for 2 h. Cultures were washed three times to remove extracellular bacteria. Infected macrophages were cultured overnight, and cytokines were added on day 1. For CFU measurements, cells were lysed with 1% Triton X-100–PBS, and lysates from quadruplicate conditions were plated in serial dilutions on Middlebrook 7H10 agar plates (Thermo Fisher Scientific) and cultured at 37°C for 21 days. Infected macrophages were treated with the following reagents: recombinant murine GM-CSF and IFN-γ (PeproTech).

### CFU reduction determinations.

To compare inhibition of bacterial growth across multiple experiments, the percent CFU reduction was calculated. One hundred percent CFU reduction on day 5 indicated complete inhibition of bacterial growth from day 1 levels, while 0% CFU reduction indicated no change in bacterial growth from untreated macrophages. The following formula was used: percent CFU reduction = 100 × {[CFU_(untreated mf day 5)_ − CFU_(treated mf day 5)_]/[CFU_(untreated mf day 5)_ − CFU_(untreated mf day 1)_]}, where “mf” is macrophages.

### Bioplex immunoassay.

Protein from lung homogenates was extracted using the Bio-Plex cell lysis kit (Bio-Rad) and filtered through a 0.2-µm filter to remove any bacteria. The concentration of GM-CSF was measured using a mouse Bio-Plex cytokine assay in accordance with the manufacturer’s instructions (Bio-Rad).

### Statistical analysis.

All data are presented as means ± SEM. Data were analyzed by one-way ANOVA (with 95% confidence intervals) and Dunnett’s posttest (for comparison against a single control) or unpaired Student’s *t* test. Analysis was performed using GraphPad Prism software.

10.1128/mBio.01514-17.2 TABLE S1  Statistical analysis of the WT and GM-CSF^−/−^ bone marrow chimeras. Download TABLE S1, PDF file, 0.02 MB. Copyright © 2017 Rothchild et al. 2017 Rothchild et al. This content is distributed under the terms of the Creative Commons Attribution 4.0 International license.
