# Supplementary material for: Role of Granulocyte-Macrophage Colony-Stimulating Factor Production by T Cells during Mycobacterium tuberculosis Infection
Source: mBio. 2017 Oct 24;8(5):e01514-17. doi: 10.1128/mBio.01514-17 (PMC5654932; doi:10.1128/mBio.01514-17)
Supplement: FIG S1 [file mbo005173557sf1.pdf]

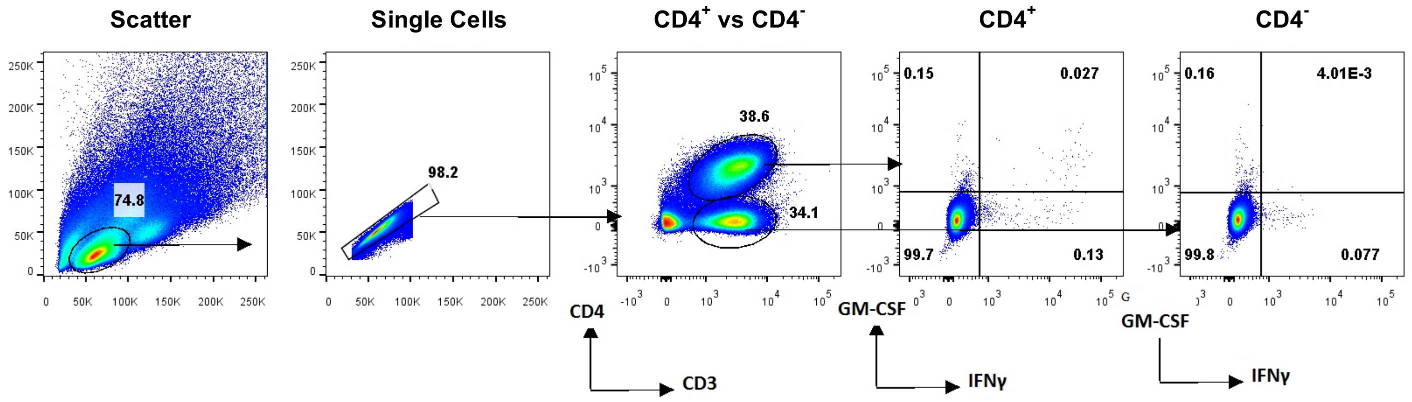

Supplemental Figure 1: **Gating strategy for human PBMC**. Lymphocytes were identified based on typical FSC and SSC pattern, and then singlets were gated to avoid cell clumps. $CD4^+$ T cells were identified based on their dual staining with antibodies specific for CD3 and CD4. $CD4^-$ T cells were defined based on positive staining for CD3 and negative staining for CD4.
