# Supplementary material for: Role of Granulocyte-Macrophage Colony-Stimulating Factor Production by T Cells during Mycobacterium tuberculosis Infection
Source: mBio. 2017 Oct 24;8(5):e01514-17. doi: 10.1128/mBio.01514-17 (PMC5654932; doi:10.1128/mBio.01514-17)
Supplement: TABLE S1 [file mbo005173557st1.pdf]

Supplemental Table 1: Statistical analysis of BM chimeras

	Experiment #1		Experiment #2		Combined	
	Mean △	Signif*	Mean △	Signif	Mean △	Signif
WT>WT vs. WT>KO	-0.92	ns	-0.89	ns	-0.90	ns
WT>WT vs. KO>WT	-0.18	ns	-0.13	ns	-0.16	ns
WT>WT vs. KO>KO	-1.71	*	-2.36	**	-2.04	****
WT>KO vs. KO>WT	0.73	ns	0.75	ns	0.74	ns
WT>KO vs. KO>KO	-0.79	ns	-1.48	ns	-1.13	*
KO>WT vs. KO>KO	-1.52	*	-2.23	**	-1.88	***

* One-way ANOVA:
